# High-Order Epistasis and Functional Coupling of Infection Steps Drive Virus Evolution toward Independence from a Host Pathway

**DOI:** 10.1128/Spectrum.00800-21

**Published:** 2021-09-01

**Authors:** Minetaro Arita

**Affiliations:** a Department of Virology II, National Institute of Infectious Diseases, Tokyo, Japan; Regional Centre for Biotechnology

**Keywords:** virus, resistance, PI4KB, OSBP, evolution, epistasis, enterovirus, evolutionary biology, virus-host interactions

## Abstract

The phosphatidylinositol-4 kinase IIIβ (PI4KB)/oxysterol-binding protein (OSBP) family I pathway serves as an essential host pathway for the formation of viral replication complex for viral plus-strand RNA synthesis; however, poliovirus (PV) could evolve toward substantial independence from this host pathway with four mutations. Recessive epistasis of the two mutations (3A-R54W and 2B-F17L) is essential for viral RNA replication. Quantitative analysis of effects of the other two mutations (2B-Q20H and 2C-M187V) on each step of infection reveals functional couplings between viral replication, growth, and spread conferred by the 2B-Q20H mutation, while no enhancing effect was conferred by the 2C-M187V mutation. The effects of the 2B-Q20H mutation occur only via another recessive epistasis between the 3A-R54W/2B-F17L mutations. These mutations confer enhanced replication in PI4KB/OSBP-independent infection concomitantly with an increased ratio of viral plus-strand RNA to the minus-strand RNA. This work reveals the essential roles of the functional coupling and high-order, multi-tiered recessive epistasis in viral evolution toward independence from an obligatory host pathway.

**IMPORTANCE** Each virus has a different strategy for its replication, which requires different host factors. Enterovirus, a model RNA virus, requires host factors PI4KB and OSBP, which form an obligatory functional axis to support viral replication. In an experimental evolution system *in vitro*, virus mutants that do not depend on these host factors could arise only with four mutations. The two mutations (3A-R54W and 2B-F17L) are required for the replication but are not sufficient to support efficient infection. Another mutation (2B-Q20H) is essential for efficient spread of the virus. The order of introduction of the mutations in the viral genome is essential (known as “epistasis”), and functional couplings of infection steps (i.e., viral replication, growth, and spread) have substantial roles to show the effects of the 2B-Q20H mutation. These observations would provide novel insights into an evolutionary pathway of the virus to require host factors for infection.

## INTRODUCTION

Enterovirus (EV) is a small nonenveloped virus with a positive-sense single-stranded RNA genome of about 7,500 nucleotides (nt). The genus *Enterovirus* in the family *Picornaviridae* consists of 15 virus species as of March 2021. Some of the members can cause severe neurological diseases, including poliovirus (PV) (*Enterovirus C*), enterovirus 71 (EV-A71) (*Enterovirus A*), and probably enterovirus 68 (EV-D68) (*Enterovirus D*).

EV infection causes membrane rearrangements in the endoplasmic reticulum in the cell (1) to form a virus-induced membranous organelle called a replication organelle (RO), which was typically observed as a double-membrane vesicle (2, 3). The phosphatidylinositol-4 kinase IIIβ (PI4KB) (4) and oxysterol-binding protein (OSBP) family I (5–8) have been discovered as the host factors required for the replication of EV. A similar host pathway (PI4KB/PI4KA and OSBP) is conserved among replications of other members of picornavirus (Aichi virus 1 and encephalomyocarditis virus) (9, 10) and of hepatitis C virus (HCV) (11–16). PI4KB is one of the four mammalian PI4 kinases (17) and produces phosphatidylinositol 4-phosphate (PI4P) mainly at the Golgi compartment. OSBP transfers cholesterol between the endoplasmic reticulum and *trans*-Golgi compartment in a PI4P-dependent manner (18–21). In EV infection, PI4KB is activated by viral proteins (2C, 2BC, 3AB, 3CD, and 3D) (22, 23) and provides PI4P on RO, which recruits OSBP (24). OSBP accumulates unesterified cholesterol on RO (8, 24) and enhances cleavage of 3AB (22, 25, 26) and development of RO (27), along with phosphatidylcholine (PC) synthesized via lipolysis (28, 29), ultimately for viral plus-strand RNA synthesis (30, 31). Membrane rearrangement caused by 2BC and 3A might support the major roles of these viral proteins in PI4KB activation (32). Resistance mutations in 3A to PI4KB/OSBP inhibitors facilitate the cleavage of 3AB and development of RO (22, 25, 27), probably via attenuated association of 3AB with the membrane and enhancement of the cleavage by 3CD protease (33, 34). Produced 3B (also known as VPg) serves as the primer for the viral RNA synthesis after uridylylation (35, 36). The PI4KB/OSBP pathway also has a role to counteract preexistent type I interferon response in PV infection (27) as well as choline/PC synthesis pathway (28).

Despite the conserved roles of the PI4K/OSBP pathway in viral replication, a virus could evolve or deadapt to replicate independently of the pathway with a few mutations *in vitro* (27). During adaptation of PV in *PI4KB*-knockout cells, four mutations were sequentially fixed in the following order: the 3A-R54W, 2C-M187V, 2B-F17L, and 2B-Q20H mutations (27). The 3A-R54W/2B-F17L mutations were essential for PI4KB/OSBP-independent replication and substantially complete resistance to the PI4KB/OSBP inhibitors; the 3A mutation conferred enhanced cleavage of 3AB and development of RO, while the 2B mutation conferred adaptation to the altered lipid environment. The 3A-R54W mutation was the prerequisite for the effects of the 2B-F17L mutation, suggesting a form of recessive epistasis in a haploid viral genome (37). In contrast, the 2B-Q20H and 2C-M187V mutations showed minor or suppressive effects on the replication, thus the importance of these mutations remained unclear.

In this study, we analyze the roles of the 2B-Q20H and 2C-M187V mutations in PI4KB/OSBP-independent infection. Quantitative analysis on the effects of the mutations in each infection step reveals essential roles of functional coupling among the infection steps and high-order recessive epistasis of the mutations.

## RESULTS

### High-order recessive epistasis of the 2B-Q20H mutation in the viral spread from the infected cells, infectivity, and viral growth in PI4KB/OSBP-independent replication.

**FIG 1:**
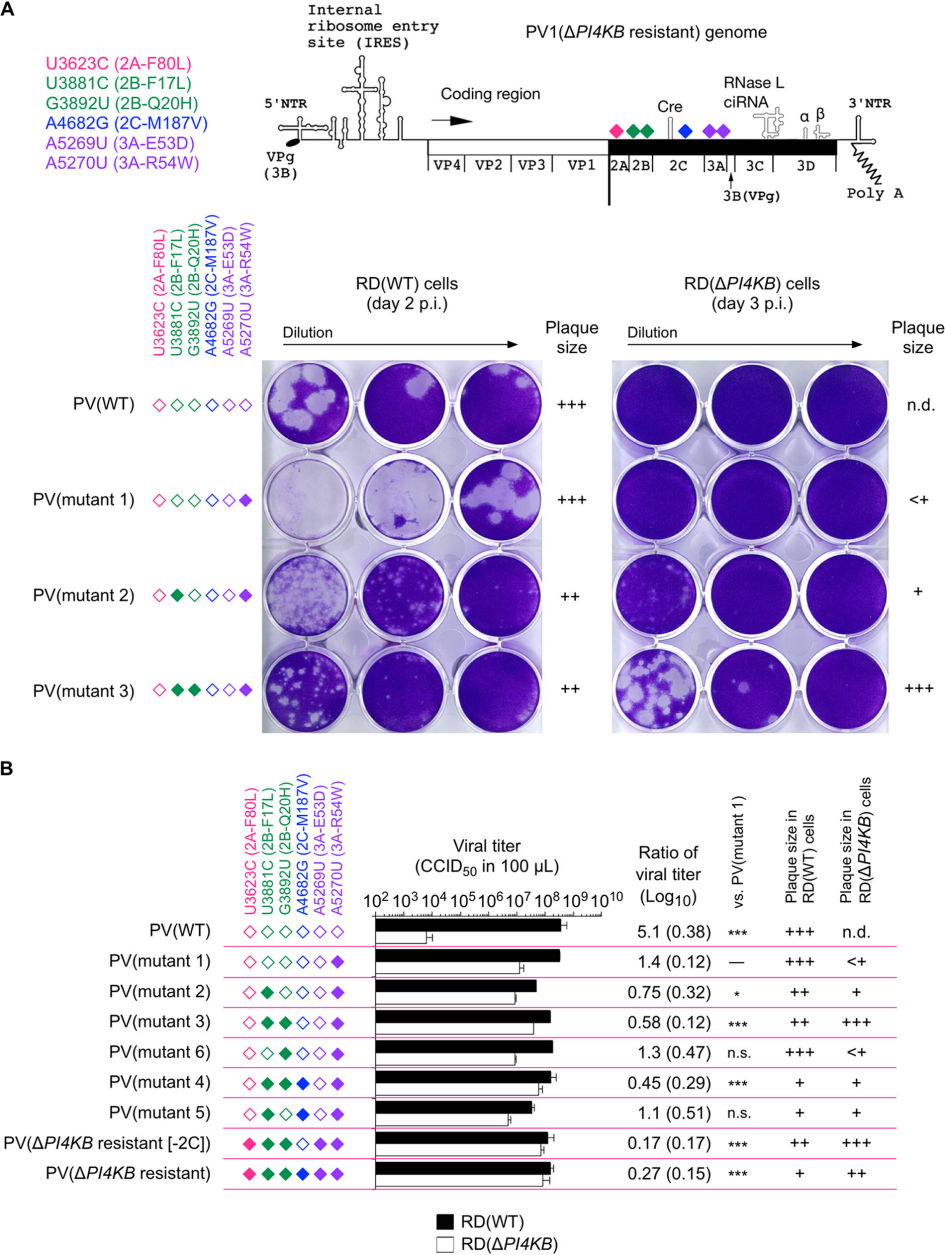
Analysis of high-order epistasis of the 2B mutations in viral spread from the infected cells in PI4KB/OSBP-independent infection. (A, Top) Schematic view of PV1(Δ*PI4KB* resistant) genome. (Bottom) Plaque phenotypes of PV mutants in RD(WT) cells or in RD(Δ*PI4KB*) cells. RD(WT) cells or RD(Δ*PI4KB*) cells were inoculated with diluted PV mutants and then stained at 2 days p.i. or at 3 days p.i., respectively. Dilution of WT and mutant 3 were 1/10^5^, 1/10^6^, and 1/10^7^. Dilution of mutants 1 and 2 were 1/10^4^, 1/10^5^, and 1/10^6^. Filled diamonds represent nucleotide mutations derived from PV(Δ*PI4KB* resistant), and open diamonds represent nucleotides of the WT. The data are representative of three independent experiments with two biological replicates. (B) Virus titer determined in RD(WT) cells or in RD(Δ*PI4KB*) cells. Ratio of virus titer represents log_10_[CCID_50_ in RD(WT) cells/CCID_50_ in RD(Δ*PI4KB*) cells]. Standard deviation is shown in parentheses. The data represent mean and standard deviation of three independent experiments with two biological replicates.

To evaluate the importance of the four mutations (3A-R54W, 2C-M187V, 2B-F17L, and 2B-Q20H mutations) in infection, we first analyzed plaque phenotypes of PV(Mahoney) mutants with the mutations in wild type (WT) and *PI4KB*-knockout RD cells—RD(WT) and RD(Δ*PI4KB*) cells, respectively (Fig. 1A). In RD(WT) cells (i.e., PI4KB/OSBP-dependent infection), the 2B-F17L and 2C-M187V mutations conferred small plaque phenotypes, while the 3A-R54W and 2B-Q20H mutations showed no effect on the phenotype (large plaque phenotypes). In RD(Δ*PI4KB*) cells (i.e., PI4KB/OSBP-independent infection), the 3A-R54W mutation conferred a small or almost invisible plaque phenotype (mutant 1). The 2B-F17L mutation could partially rescue the sizes of the PV(3A-R54W) mutant in RD(Δ*PI4KB*) cells (mutant 2). The 2C-M187V mutation conferred small plaque phenotypes in RD(Δ*PI4KB*) cells as well as in RD(WT) cells (mutants 4, 5, and Δ*PI4KB* resistant) (see Fig. S1 in the supplemental material). PV mutants with the 3A-R54W/2B-F17L/2B-Q20H mutations [mutant 3 and Δ*PI4KB*-(2C)] substantially restored the plaque phenotypes in RD(Δ*PI4KB*) cells, although the spread was slower than that in RD(WT) cells. Interestingly, the 2B-Q20H mutation could not confer a large plaque phenotype in the absence of the 2B-F17L mutation in RD(Δ*PI4KB*) cells (mutant 6) (see Fig. S2 in the supplemental material), suggesting that the 2B-F17L mutation acts as a prerequisite for the effect of the 2B-Q20H mutation on the viral spread from the infected cells.

**FIG 2:**
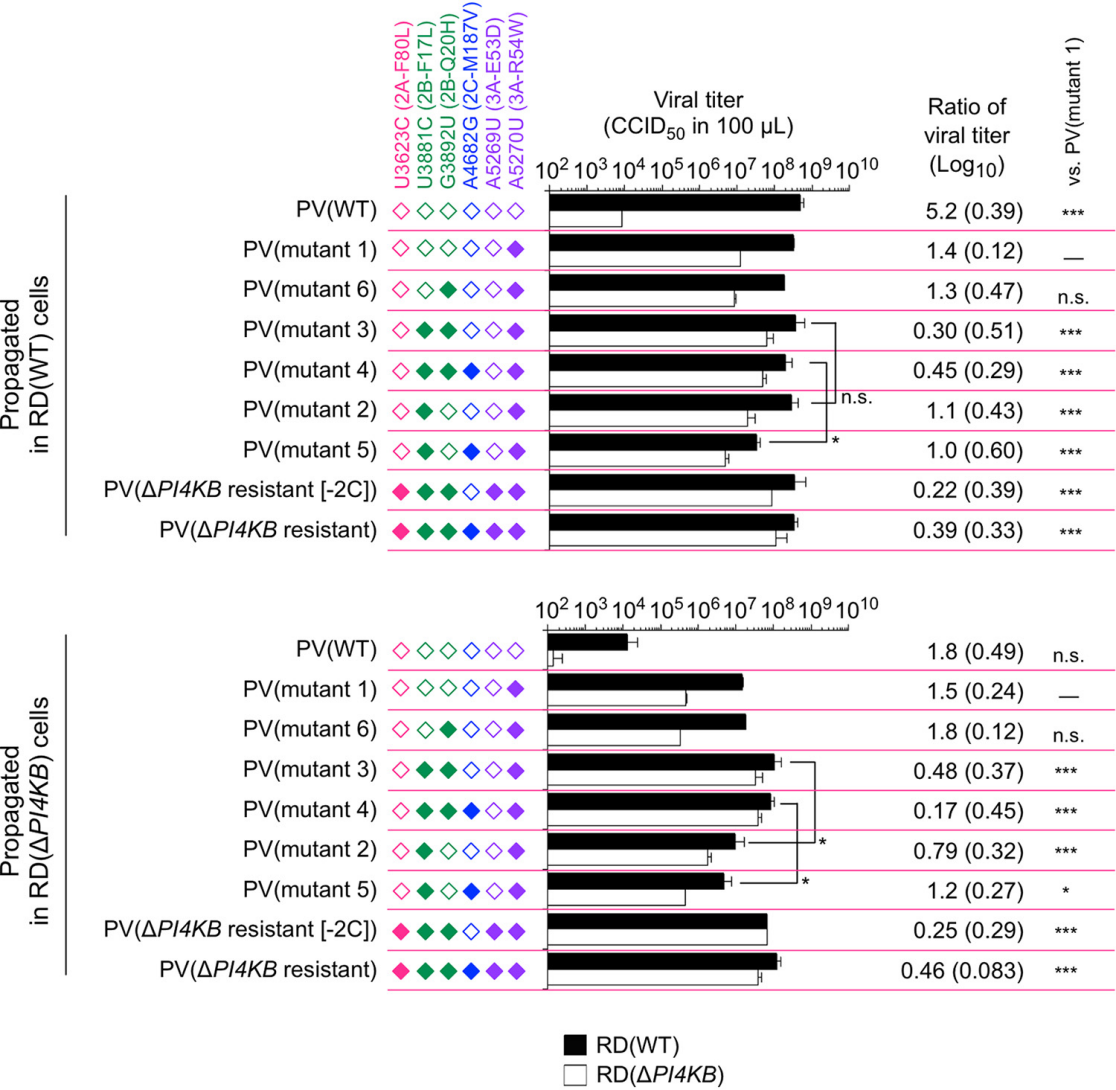
Analysis of high-order epistasis of the 2B mutations in infectivity and viral growth in PI4KB/OSBP-independent infection. RD(WT) cells or RD(Δ*PI4KB*) cells were infected with PV mutants at a multiplicity of infection (MOI) of 10 and then harvested at 24 h p.i. Virus titer was measured in RD(WT) cells or in RD(Δ*PI4KB*) cells. Virus titer and the ratio of virus titer measured in RD(WT) cells to that in RD(Δ*PI4KB*) cells are shown. Standard deviation is shown in parentheses. Filled diamonds represent nucleotide mutation derived from PV(Δ*PI4KB* resistant), and open diamonds represent nucleotides of the WT. The data represent mean and standard deviation of three independent experiments with two biological replicates. n.s., not significant.

Next, we analyzed the relative infectivity of the PV mutants in RD(Δ*PI4KB*) cells (Fig. 1B) and viral growth (Fig. 2). The 3A-R54W mutant (mutant 1) showed significantly increased infectivity in RD(Δ*PI4KB*) cells in contrast to the parental strain [difference between viral titers in RD(WT) and RD(Δ*PI4KB*) cells was only 1.4 Log_10_ versus 5.1 Log_10_ in PV(WT)]. The 2B-F17L mutation conferred enhanced infectivity on the 3A-R54W mutant (mutant 2; the difference in viral titers was 0.75 Log_10_), while the 2C-M187V mutation showed no effect. The 2B-Q20H mutation conferred enhanced infectivity in the 3A-R54W/2B-F17L mutants [mutants 3, 4, Δ*PI4KB*-(2C), and Δ*PI4KB* resistant; the differences in viral titers were 0.17 to 0.58 Log_10_] but not in the mutants without the 2B-F17L mutation (mutants 5 and 6).

After propagation in RD(WT) cells, the titers of the PV mutants were similar (Fig. 2), except for the PV(2C-M187V) mutant (mutant 5). Growth of the PV mutants in RD(Δ*PI4KB*) cells were generally suppressed compared to that in RD(WT) cells; however, the 3A-R54W/2B-F17L/2B-Q20H mutants [mutants 3, 4, Δ*PI4KB*-(2C), and Δ*PI4KB* resistant] showed similar orders of titers after propagation in RD(Δ*PI4KB*) cells. The 2B-Q20H mutation conferred enhanced growth on the 3A-R54W/2B-F17L mutants as well as infectivity in RD(Δ*PI4KB*) cells, while the 2C-M187V mutation showed no effect on the titers. The ratio of viral titers of PV(WT) was significantly reduced after propagation in RD(Δ*PI4KB*) cells and was similar to that of the 3A-R54W mutant (mutant 2), probably because of the rapid emergence of a small population of resistant mutants (27). The ratios for other mutants remained similar before and after propagation in RD(Δ*PI4KB*) cells. Collectively, these results suggested that the 2B-Q20H mutation, but not the 2C-M187V mutation, could confer enhanced infectivity, viral growth, and viral spread in PI4KB/OSBP-independent infection via recessive epistasis between the 3A-R54W/2B-F17L mutations.

### Roles of the 2B mutations in viral replication, cell death, and infectivity in PI4KB/OSBP-independent replication.

**FIG 3:**
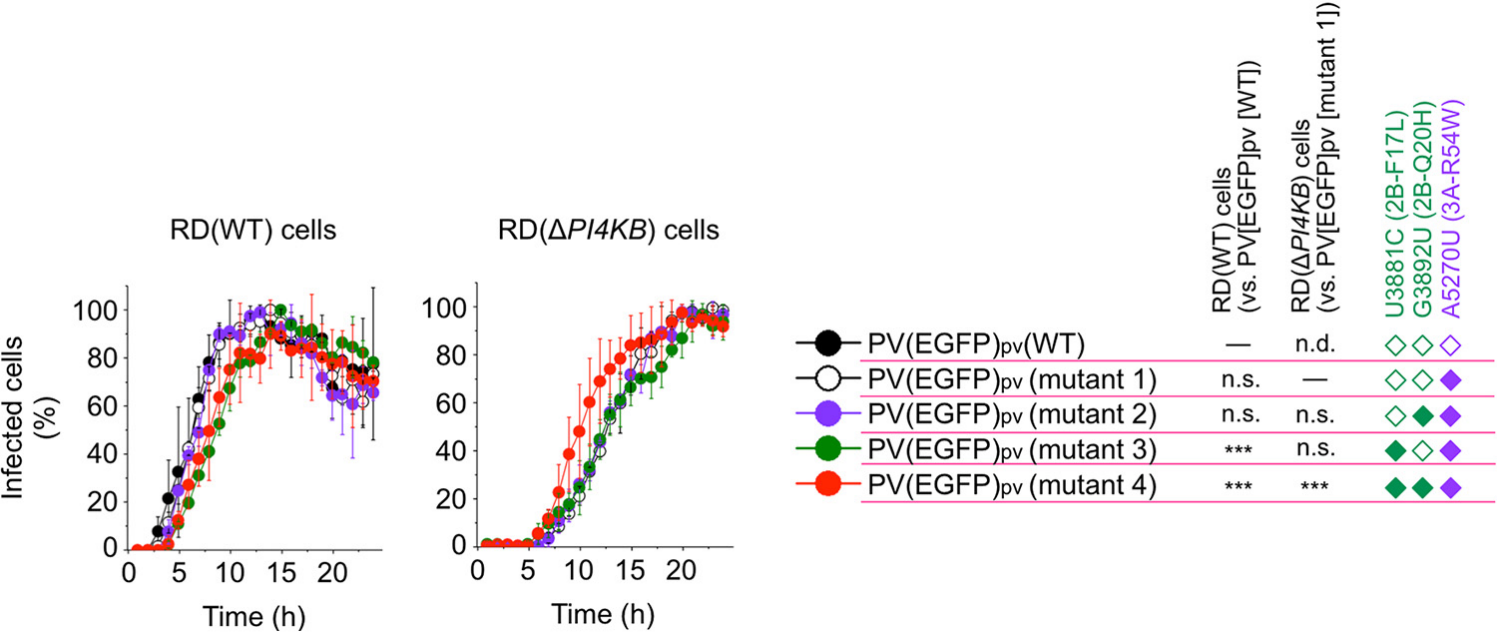
Replication kinetics of PV(EGFP)_pv_ mutants in PI4KB/OSBP-independent infection. The cells were infected with PV(EGFP)_pv_ at an MOI of 10 and then observed for 24 h by a time-lapse fluorescence microscopy (LCV110; Olympus). Percentages of infected cells are shown. Maximum number of the infected cells observed in each infection was taken as 100%. Kinetics of the infection was analyzed by a paired *t* test. n.d., not detectable. The data represent mean and standard deviation of two independent experiments with two biological replicates.

In a previous study, we found that the 2B-F17L/2B-Q20H mutations could confer an enhanced replication level in PI4KB/OSBP-independent replication (27), but the roles of individual mutations remained to be clarified. To dissect the roles of the 2B mutations, we performed a single-cell analysis on the infection of PV pseudovirus (PV_pv_) with enhanced green fluorescence protein as the reporter [PV(EGFP)_pv_] (see Fig. S3 in the supplemental material). We first characterized replication kinetics of PV(EGFP)_pv_ mutants (Fig. 3; see also Videos S1 to S4 in the supplemental material). The 2B-F17L mutants (mutants 3 and 4) showed delayed replication in RD(WT) cells (plateau at about 14 hours postinfection [h p.i.] versus 12 h p.i. of WT, mutant 1, and mutant 2), while the 2B-Q20H mutation did not affect the kinetics. In RD(Δ*PI4KB*) cells, the 2B-Q20H mutation conferred a significantly enhanced replication rate in the presence of the 2B-F17L mutation (plateau of mutant 4 at about 20 h p.i. versus 22 h p.i. of mutants 1, 2, and 3). These results suggest that the 2B-Q20H mutation could confer an enhanced replication rate in PI4KB/OSBP-independent infection via recessive epistasis between the 3A-R54W/2B-F17L mutations.

**FIG 4:**
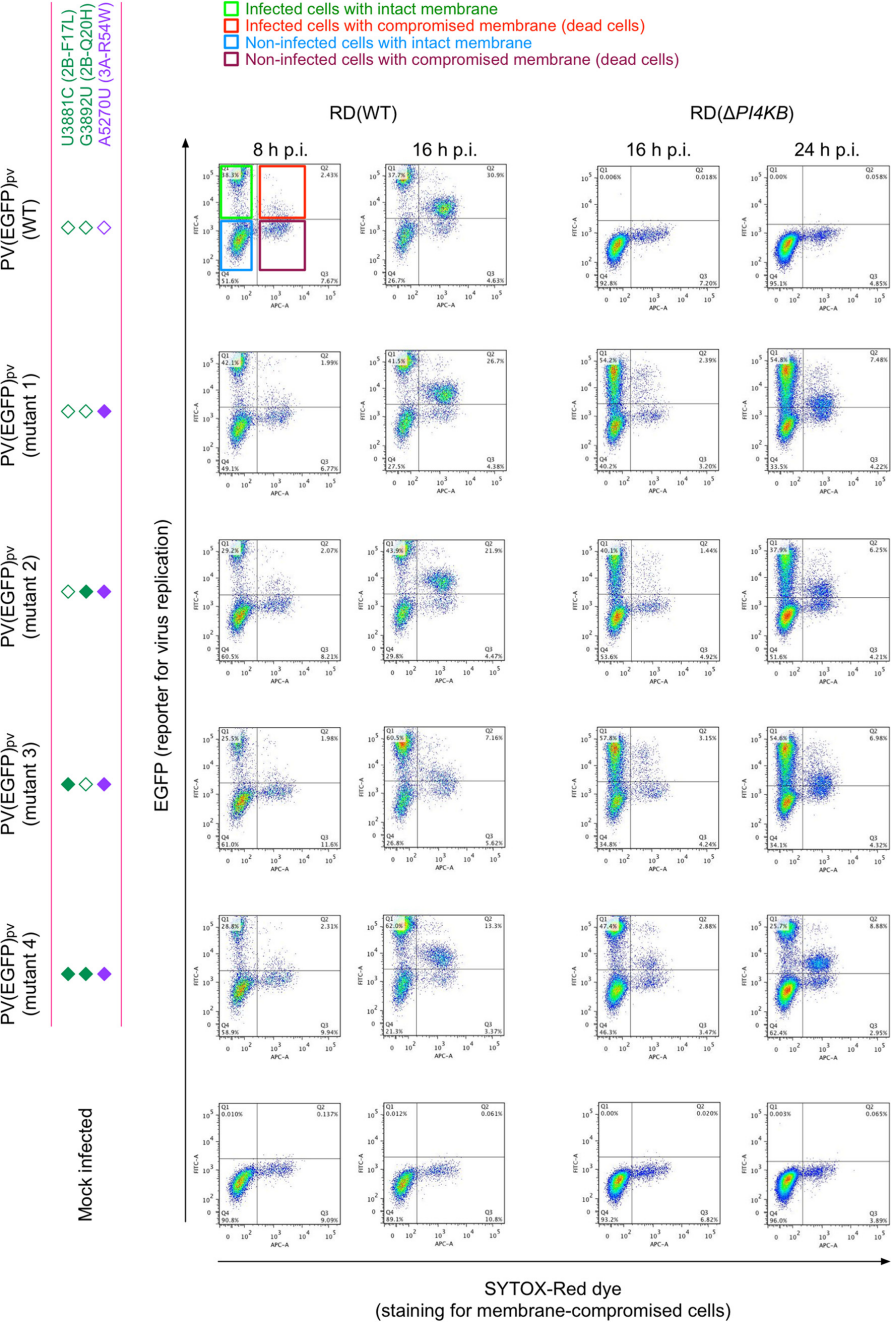
Flow cytometry analysis of viral replication level, cell death, and infectivity in PI4KB/OSBP-independent infection. Flow cytometry analysis of RD(WT) cells or RD(Δ*PI4KB*) cells infected with PV(EGFP)_pv_ mutants at an MOI of 0.5 at 8, 16, or 24 h p.i. is shown. Infected cells were identified by EGFP produced by the infection. Membrane-compromised cells (dead cells) were identified by a membrane-impermeable dye for nuclei staining (SYTOX red dye). The data are representative of two independent experiments with three biological replicates.

**FIG 5:**
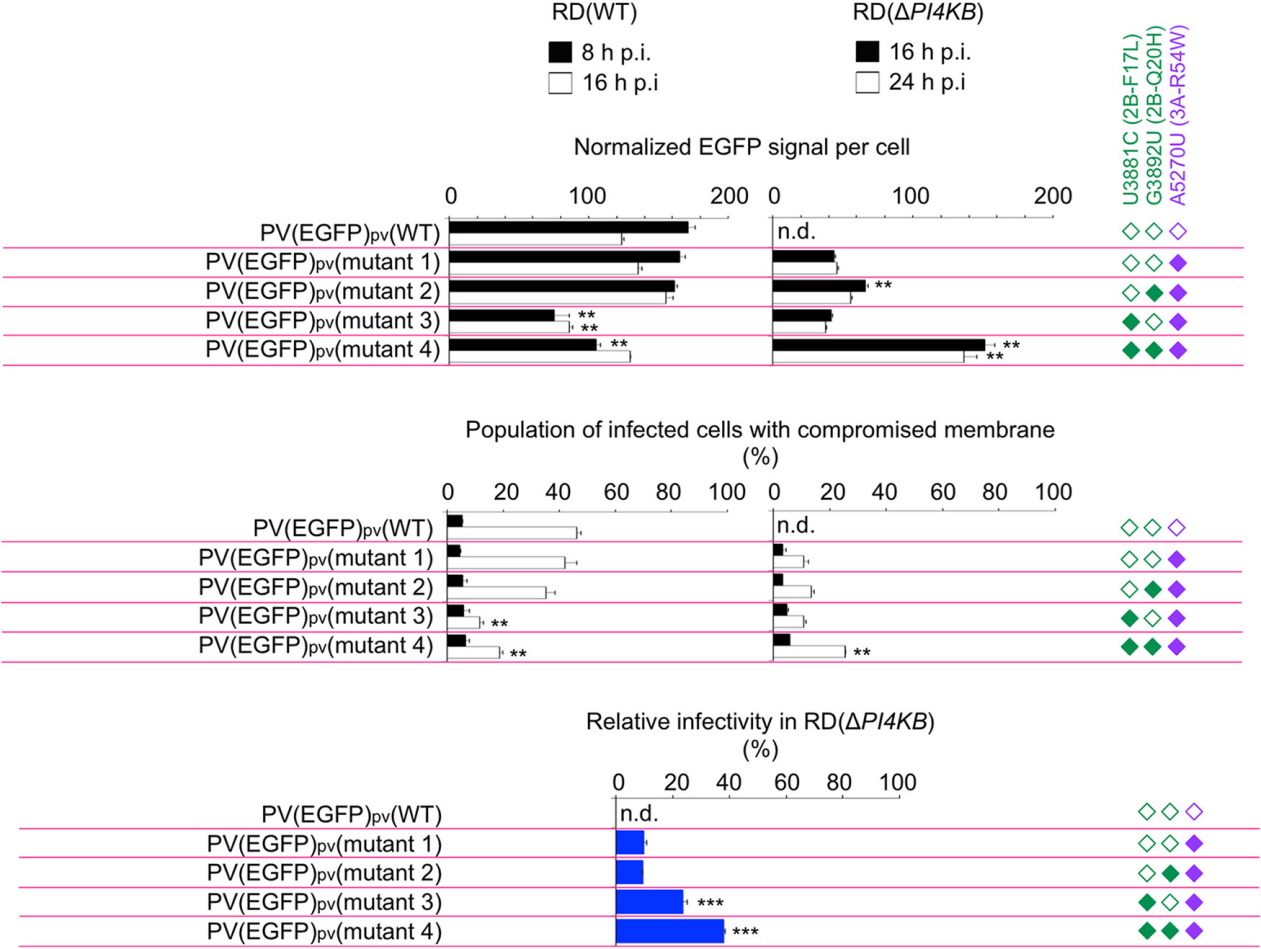
Quantification of effects of the mutations on viral replication level, cell death, and infectivity in PI4KB/OSBP-independent infection. Quantification of the data shown in Fig. 3 is shown. Normalized EGFP signal per cell (EGFP signal in noninfected cells is taken as 1), population of infected cells with compromised membrane (total number of infected cells is taken as 100%), and relative infectivity in RD(Δ*PI4KB*) cells (total number of infected RD(WT) cells is taken as 100%) are shown. For the measurement of the relative infectivity in RD(Δ*PI4KB*) cells, infected RD(WT) cells and infected RD(Δ*PI4KB*) cells were counted at 16 h p.i. and 20 h p.i., respectively. The data represent mean and standard deviation of two independent experiments with three biological replicates. Statistical significance to PV(EGFP)_pv_(mutant 1) infection was analyzed. n.d., not detectable.

Next, we performed single-cell analysis of the infection of PV(EGFP)_pv_ mutants in terms of the integrity of the plasma membrane, which could affect lytic viral spread, and viral replication level by fluorescence intensity of EGFP and by staining with a membrane-impermeable dye (SYTOX red dye), respectively (Fig. 4 and 5). In RD(WT) cells, the maximum replication levels of the PV(EGFP)_pv_(2B-F17L) mutants (mutants 3 and 4) were reduced (about 2-fold reduction compared to that of mutant 1) (Fig. 4 and 5). Impairment of the membrane integrity of RD(WT) cells could be observed at 16 h p.i. and showed a positive correlation with the replication level. In RD(Δ*PI4KB*) cells, significant enhancements in the replication level and infectivity were observed for the 2B-F17L/2B-Q20H mutant (mutant 4; about 4-fold enhancement compared to that of mutant 1) but not for the 2B-Q20H mutant without the 2B-F17L mutation (mutant 2). The 2B-F17L mutation did not confer enhanced replication level but conferred enhanced infectivity in RD(Δ*PI4KB*) cells (mutant 3). Impairment of the membrane integrity of RD(Δ*PI4KB*) cells showed a positive correlation with the replication level as observed in RD(WT) cells. These results suggested that there are positive correlations among the replication level, the membrane integrity, and the infectivity in PI4KB/OSBP-independent infection conferred by the 2B-Q20H mutation and that the 2B mutations have two distinct functions; the 2B-F17L mutation confers enhanced infectivity in PI4KB/OSBP-independent infection without affecting the replication level, while the 2B-Q20H mutation confers enhanced replication level and infectivity only via recessive epistasis between the 3A-R54W/2B-F17L mutations.

### The ratio of viral plus-strand RNA to the minus-strand RNA in PI4KB/OSBP-independent infection.

**FIG 6:**
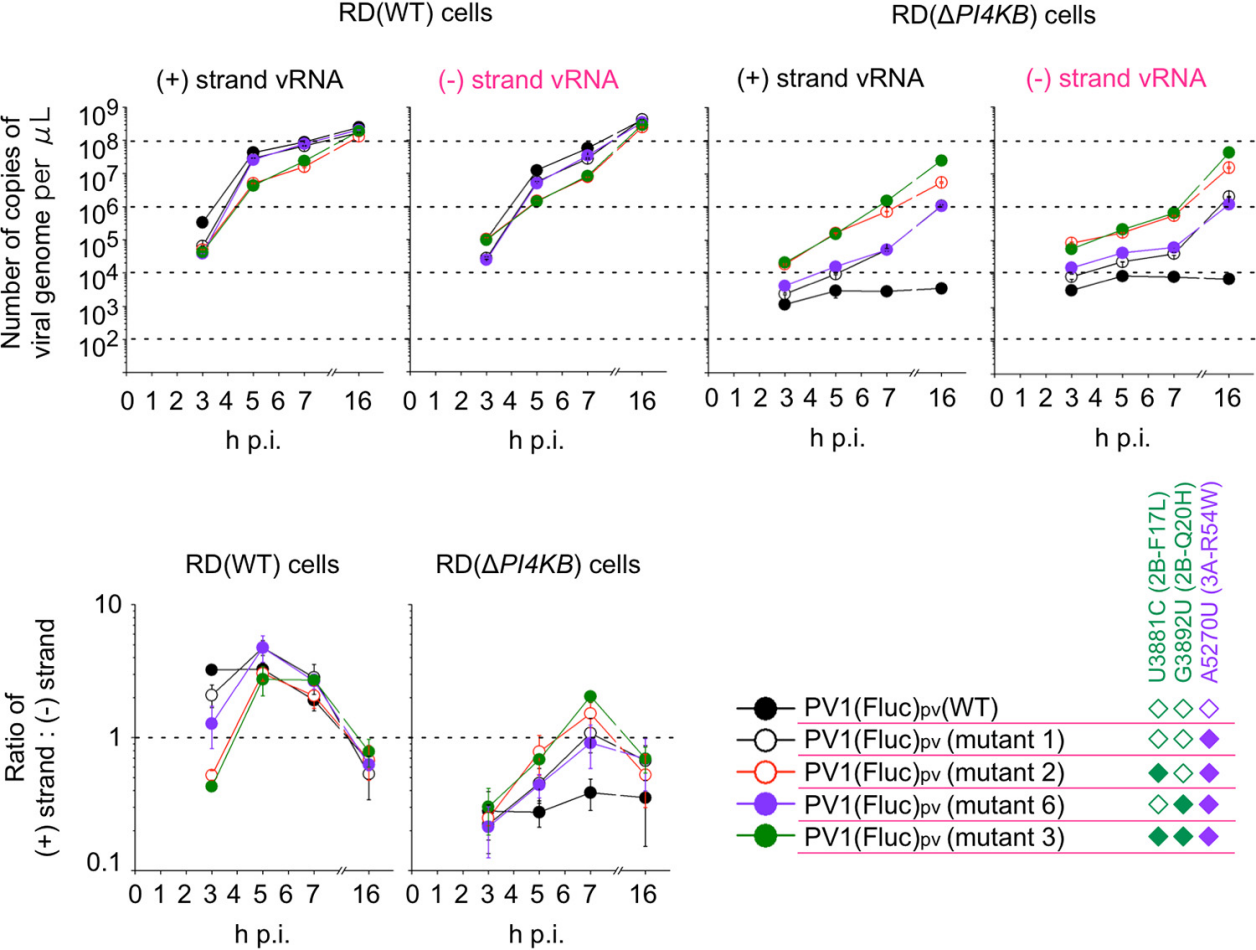
Ratio of viral plus-strand RNA to the minus-strand RNA in PI4KB/OSBP-independent infection. (Top) Number of copies of viral RNA in the cells infected with PV(Fluc)_pv_ mutants determined by strand-specific RT-PCR is shown. (Bottom) Ratio of the plus-strand RNA and minus-strand RNA at indicated time after the infection is shown. The data represent mean and standard deviation of two independent experiments with three biological replicates.

Previous reports suggested that PI4KB inhibitors suppress the formation of viral replication complex for viral plus-strand RNA synthesis (30, 31). Therefore, we quantified the ratio of viral plus-strand RNA to the minus-strand RNA in PI4KB/OSBP-independent infection and analyzed the effects of the mutations on the ratio (Fig. 6). We developed a modified strand-specific real-time reverse transcription-PCR (RT-PCR) assay targeting the firefly luciferase gene in the replicon to analyze the ratio in a single-cycle infection by PV_pv_ with firefly luciferase as the reporter [PV(Fluc)_pv_] (see Table S1 in the supplemental material). The ratio of the signal of false-negative minus-strand RNA to that of the true-positive plus-strand RNA was 0.00075. The sensitivity of the minus-strand RNA detection was about 1/10 of that of plus-strand RNA detection, resulting in 1/140 of the true-positive plus-strand RNA detected as false-positive negative-strand RNA; i.e., the minus-strand RNA more than 1/140 of the plus-strand RNA could be detected as true-positive minus-strand RNA.

The ratio of the RNAs in PV(Fluc)_pv_-infected cells was determined in a range of 0.21 to 5.6 (Fig. 6). Therefore, the minus-strand RNA in the infected cells was detected above the specificity limit in this system. The ratio of the RNAs showed positive correlations with replication level and rate in both RD(WT) cells and RD(Δ*PI4KB*) cells and peaked at the mid-log phase of the replication followed by a decrease. In RD(WT) cells, the 2B-F17L mutation conferred attenuated replication and reduced ratio of the RNAs compared to those in PV(Fluc)_pv_(WT) infection. In RD(Δ*PI4KB*) cells, the 2B-Q20H mutation conferred an enhanced replication level concomitantly with the increased ratio of the RNAs only in the presence of the 3A-R54W/2B-F17L mutations. The minus-strand RNA could be detected in RD(Δ*PI4KB*) cells infected with PV(Fluc)_pv_(WT), but no increase of the plus-strand RNA was observed, supporting that the PI4KB/OSBP pathway is required for plus-strand RNA synthesis. These results suggested that there is a positive correlation between the replication rate/level and the ratio of the plus-strand RNA to the minus-strand RNA, which caused the dynamic behavior of the ratio of the RNAs in PV replication. The 3A and 2B mutations could confer specific enhancement of the replication level and rate in PI4KB/OSBP-independent infection concomitantly with an increased ratio of the RNAs.

## DISCUSSION

Mutations in 3A of enterovirus have been identified as the major determinants for resistance to PI4KB/OSBP inhibitors (38) but confer only partial resistance (22, 39). In this study, we found that viral spread from infected cells is the bottleneck of the PV 3A-R54W mutant in PI4KB/OSBP-independent infection (Fig. 1). This defect could be rescued with the 2B mutations via multitiered recessive epistasis. The 2B-F17L mutation enhanced the infectivity without enhancing the replication rate or level in PI4KB/OSBP-independent infection via recessive epistasis between the 3A-R54W mutation. The 2B-Q20H mutation enhanced the replication rate/level and viral growth via recessive epistasis between the 3A-R54W/2B-F17L mutations (Fig. 2 to 5). Delayed RO formation under PI4KB inhibition has been observed in infection of a CVB3 3A mutant (25), thus enhancement of the replication rate might serve as a specific determinant for evolution toward PI4KB/OSBP-independent infection. A positive correlation between the population of PV-infected cells with compromised membrane and the replication rate/level suggested that the 2B-Q20H mutation could enhance lytic viral spread. In PV infection, the lytic and nonlytic pathways have been observed (40–42). The amount of virus released via the nonlytic pathway is rather minor (about 1/1,000 of the intracellular virus at 5 h p.i.) (40). The observed enhancement of viral spread conferred by the 2B-F17L mutation might be caused by the increased number of the infected cells in the second round of infection or by enhancement of the nonlytic pathway. At least two different mechanisms seemed to support the enhanced viral spread conferred by the 2B mutations.

In contrast to the 2B mutations, the 2C-M187V mutation did not confer any enhancing effect but rather suppressed the replication rate and viral spread (Fig. 2; see also Fig. S1 in the supplemental material). Amino acid (aa) 187 of 2C can be involved in guanidine dependence/resistance of PV (43) via ATPase and/or helicase activity (44, 45). The mechanism and role of the suppressive effects remain unclear, but this mutation might have served as a 'trickster', like Puck in Shakespeare's play, for the acquisition of the 2B-Q20H mutation to rescue the infection.

Functional coupling between infection steps (translation, replication, and packaging/assembly) has been proposed as a mechanism for proofreading production of an infectious virus (46, 47). Quantitative analysis revealed that the 2B-Q20H mutation confers moderate effects in each step via recessive epistasis, 3-fold enhancement in the replication levels and 4-fold enhancement in the infectivity (Fig. 4 and 5). Similar epistasis of the 2B-Q20H mutation observed in viral spread suggested a direct relationship between these steps. This indicated that the minor enhancing effects in the replication step ultimately caused substantial enhancement in viral spread in the evolution.

Adaptation of PV in PI4KB/OSBP-independent infection reached a plateau after five passages with four mutations fixed (27), suggesting that the roles of the PI4KB/OSBP pathway in the infection could be largely defined in three steps with the 3A-R54W/2B-F17L/2B-Q20H mutations. The replication rate/level and infectivity were not fully restored (about 30% to 40% of those in PI4KB/OSBP-dependent infection), consistent with observation in experimental evolution of an RNA virus with double mutations (48). Evolution of PV toward independence from host factors GBF1 and HSP90 have been reported, which are required for viral RNA replication and folding of viral capsid proteins, respectively (49, 50). PV resistance to a GBF1 inhibitor is conferred by two mutations in viral 2C and 3A in an additive manner (51). The resistance to an HSP90 inhibitor could be conferred by several mutations in the capsid proteins by modulating stability and aggregation, while epistasis among the mutations remained to be elucidated (52). Generally, the prediction of the effects of high-order epistasis is impossible (53; reviewed in reference 54). Identified multitiered recessive epistasis in PV evolution would be helpful to decipher putative epistasis in PI4K/OSBP-independent picornaviruses (8, 55–57) embedded in divergent evolution. The relationship between the modes of epistasis and the roles of host factors remains to be further elucidated.

The limitations of our study include undefined roles of 2B/2BC in the infection; observed epistasis and functional coupling could be explained with defined roles at a molecular level. The 2B/3A mutations could confer increased ratio of viral plus-strand RNA to the minus-strand RNA in PI4KB/OSBP-independent replication; however, the ratio of the RNAs showed dynamic behavior in PV replication consistent with a previous report (58) and thus was supported by several factors, including the replication rate/level and the PI4KB/OSBP pathway (Fig. 6). Specific roles of the PI4KB/OSBP pathway in the plus-strand RNA synthesis among the factors remain to be elucidated. For the detection of the minus-strand RNA extracted from infected cells, a denaturing step of the RNA (at 95°C for 5 min) and RT reaction at a high temperature (at 50°C) were essential but were not required for that of *in vitro* synthesized minus single-stranded RNA. The minus-strand RNA exists only as a form of partial or complete double-stranded RNAs (dsRNA) (known as replicative intermediate or replicative form, respectively) (59). In our attempts, we could not reconstitute partial or complete double-stranded RNAs *in vitro* that could recapitulate the reactivity of the minus-strand RNA extracted from the infected cells. In PV replication, generally, the ratio of the RNAs was about 20 to 70 (58, 60) and thus was higher than that determined in the range of 0.21 to 5.6 in this study. Positive control for the minus-strand RNA that could recapitulate that in the infected cells would give the absolute ratio of the plus-strand RNA to the minus-strand RNA in the current system.

**FIG 7:**
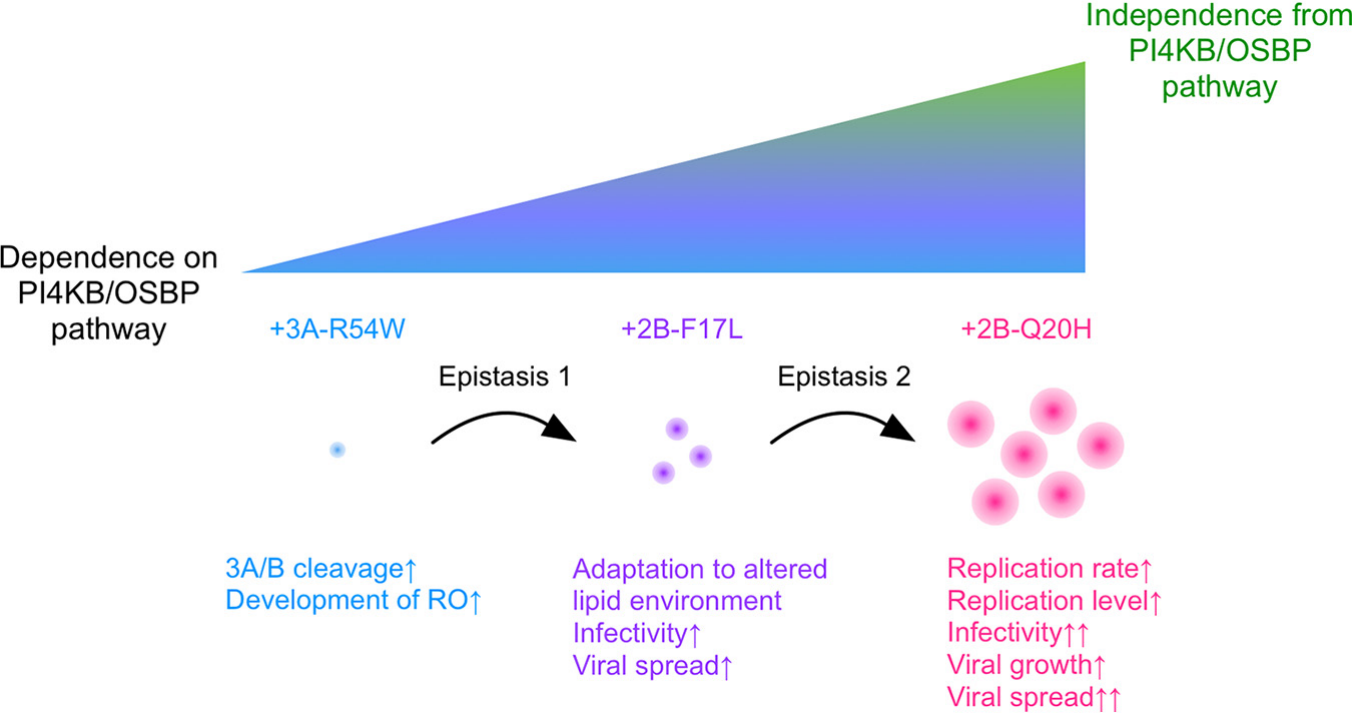
Model of the PV evolutionary pathway toward independence from the host PI4KB/OSBP pathway.

This work reveals the essential roles of functional coupling and high-order, multitiered epistasis conferred by mutations in PI4KB/OSBP-independent infection (Fig. 7). Viral spread is the bottleneck of the PV 3A-R54W mutant in infection and is rescued by the 2B-F17L/2B-Q20H mutations. The effects of the 2B mutations are supported by epistasis with the 3A mutation, which show direct links among the replication rate/level, infectivity, growth, and spread and provide a piece of evidence for a substantial role of functional couplings in the evolution. These may contribute to our fundamental understanding of the role of host factors in viral evolutionary pathways and to the strategy to overcome viral resistance against host-targeting antivirals.

## MATERIALS AND METHODS

### Cells.

RD(WT) cells (human rhabdomyosarcoma cell line), HEK293 cells (human embryonic kidney cells), and a *PI4KB*-knockout RD cell line [RD(Δ*PI4KB*)] were cultured as monolayers in Dulbecco’s modified Eagle medium (DMEM) supplemented with 10% fetal calf serum (FCS). For plaque assay, RD(WT) cells or RD(Δ*PI4KB*) cells were cultured in Eagle’s minimum essential medium (EMEM) supplemented with 10% FCS.

### Viruses.

PV mutants were obtained by RNA transfection of corresponding RNA transcripts to RD(WT) cells, which were prepared by using a T7 RiboMAX Express large-scale RNA production system (Promega; product number P1320) with DraI-linearized infectious clones of PV(Mahoney) as the templates. RNA transcripts were transfected into a monolayer of RD cells in 24-well plates (Falcon) using a Lipofectamine MessengerMAX reagent (Invitrogen; catalog number LMRNA015), followed by incubation at 37°C in 10% FCS-DMEM (1 ml per well). The cells were harvested at 24 h posttransfection (p.t.) or at 48 h p.t. when all of the cells showed the cytopathic effect and were then stored at −20°C.

### General methods for molecular cloning.

Escherichia coli strain XL10-Gold (Stratagene) was used for the preparation of plasmids. Ligation of DNA fragments was performed using an In-Fusion HD cloning kit (Clontech). PCR was performed using KOD Plus DNA polymerase (Toyobo). DNA sequencing was performed using a BigDye Terminator v3.1 cycle sequencing ready reaction kit (Applied Biosystems) and then analyzed with a 3500xL genetic analyzer (Applied Biosystems).

### Plasmids. (i) Infectious clones of PV(Mahoney) mutants.

Infectious clones of PV were constructed by replacing the firefly luciferase-coding region of PV replicons with the capsid-coding region of the type 1 PV(Mahoney) strain (GenBank accession number NM_V01149). cDNA of the PV capsid-coding region was amplified by PCR using pMah-SacI (61) as the template with primer set 1. cDNAs of PV replicons were amplified by PCR using each replicon plasmid as the template with primer set 2.

Primer set 1
5′-GACAATTGTATCATAATGGGaGCTCAGGTTTCATCACAGAAAG-3′
5′-TTGGTGTCCGAATCCATATGTGGTCAGATCCTTGGTGGAGAGG-3′
Primer set 2
5′- TATGATACAATTGTCTGATTGAAATAACTG -3′
5′- GGATTCGGACACCAAAACAAAGCGGTGTAC-3′

### (ii) EGFP-encoding PV(Mahoney) replicon.

EGFP-encoding PV replicon was constructed by PCR by deleting the capsid-coding region using pEGFP-Mah as the template (62) with primer set 3. An EcoRI site in the 5′NTR of this replicon was removed by PCR as well as a firefly luciferase-encoding PV replicon (63). Mutations of PV(Δ*PI4KB* resistant) were introduced into EGFP-encoding PV replicon by site-directed mutagenesis (27, 64).

Primer set 3
5′-GCTCACCACATATGGATTCGGACACCAAAACAAAGCGGTG-3′
5′-CCATATGTGGTGAGCCCCTTCTTGTACAGCTCGTCCATGC-3′

### (iii) Preparation and titration of EGFP-encoding PV_pv_[PV(EGFP)_pv_].

PV(EGFP)_pv_ was prepared as previously reported for luciferase-encoding PV_pv_ (65). A 6-well plate (Falcon) with a 10% confluent monolayer of HEK293 cells was transfected with 2 μg of PV(Mahoney) capsid expression vectors per well using *Trans*IT-PRO transfection kit (Mirus). The cells were incubated at 37°C in 2 ml DMEM supplemented with 10% FCS per well for 24 h. RNA transcripts of PV replicons were obtained using a RiboMAX large-scale RNA production system T7 kit (Promega) with DraI-linearized plasmids of PV replicons. RNA transcripts were transfected into the monolayer of HEK293 cells transiently expressing the capsid proteins at 24 h posttransfection using Lipofectamine MessengerMAX reagent (Invitrogen). Cells were harvested at 24 h posttransfection of the RNA transcripts and then stored at −20°C. Infectious units (IU) of PV(EGFP)_pv_ stock solution were determined by counting the number of infected cells by flow cytometry. RD(WT) cells or RD(Δ*PI4KB*) cells were infected by PV(EGFP)_pv_ at a multiplicity of infection (MOI) of approximately 0.2 at 37°C and then collected at 16 h postinfection (p.i.). The cells were stained by using SYTOX red dead cell stain (1:1,000; Invitrogen; catalog number S34859) and then analyzed by flow cytometry to detect infected cells that express EGFP.

### Titration of virus.

The titer of PV was determined by measuring 50% cell culture infectious dose (CCID_50_) by the microtitration assay. RD(WT) cells or RD(Δ*PI4KB*) cells were seeded into 384-well plates (Greiner Bio-One; 781182) (5 × 10^5^ cells per well in 20 μl of 10% FCS-DMEM) and then incubated at 37°C for 1 day. The cells were inoculated with 8 μl per well of diluted virus solutions (10-fold serial dilution from 1/10^2^ to 1/10^9^) and then incubated at 37°C for 6 days and observed for cytopathic effect (CPE). The value of CCID_50_ was calculated according to the Behrens-Kärber method (66).

### Plaque assay.

RD(WT) cells or RD(Δ*PI4KB*) cells were seeded into 12-well plates (Falcon; product number 353043) (8 × 10^5^ cells per well in 500 μl of 10% FCS-DMEM) and then incubated at 37°C for 1 day. The medium of the cells was removed and changed to 10% FCS-EMEM (500 μl per well) before inoculation of virus. The cells were inoculated with diluted virus solutions (8 μl per well) and then incubated at room temperature for 20 min, then at 37°C for 2 h. After incubation, 10% FCS-EMEM containing 0.64% Avicel RD-581 (FMC corporation) was added to the cells (500 μl per well). The cells were incubated at 37°C for 2 or 3 days and then fixed and stained with 10% formaldehyde in phosphate-buffered saline containing 0.5% crystal violet.

### Strand-specific real-time RT-PCR targeting firefly luciferase gene in PV replicon.

RD(WT) cells or RD(ΔPI4KB) cells (5.6 × 10^4^ cells per well in 50 μl medium) in 96-well plates (Corning Inc.; product number 3595) were inoculated with 50 μl of PV_pv_ at an MOI of 2 and then incubated at 37°C for 3, 5, 7, or 16 h. After the incubation, the cells were washed, replenished with 50 μl of medium, and then stored at −20°C until RNA purification.

Viral RNA was purified from the cells by using a High Pure viral RNA purification kit (Roche; product number 11858882001). Strand-specific real-time RT-PCR was performed as described previously (60, 67, 68), with modifications to target firefly luciferase gene in PV replicon genome and to increase the detection limit of viral minus-strand RNA and the strand specificity. Primers used for strand-specific real-time RT-PCR targeting firefly luciferase gene are as follows:

+strandFluc-RT: reverse transcription (RT) for plus/positive strand
5′-GGCCGTCATGGTGGCGAATAACAATTGTTCCAGGAACCAGGGC-3′
−strandFluc-RT: RT for minus/negative strand
5′-GGCCGTCATGGTGGCGAATAAACGCCAAAAACATAAAGAAAGGCCCG-3′
+strandFluc_For: real-time PCR for plus/positive strand with Tag primer
5′-ACGCCAAAAACATAAAGAAAGG-3′
−strandFluc_Rev: real-time PCR for minus/negative strand with Tag primer
5′-CAATTGTTCCAGGAACCAGGGCGTATC-3′
Tag: real-time PCR for both strands
5′-GGCCGTCATGGTGGCGAATAA-3′

Single-stranded RNAs of firefly luciferase-encoding PV replicons with plus/positive or minus/negative polarity were used for RNA standards for quantification, which were produced and purified by using a T7 RiboMAX Express large-scale RNA production system T7 kit (Promega; product number P1320) with corresponding cDNAs with T7 promoter and a NucleoSpin RNA clean-up kit (Macherey-Nagel; product number 740948.250), respectively.

Viral RNA was reverse transcribed by using a PrimeScript RT reagent kit (for real time) (TaKaRa; catalog number RR037A) with primers +strandFluc-RT (for detection of plus/positive strand viral RNA) or −strandFluc-RT (for detection of minus/negative strand viral RNA). Viral RNA (0.5 μl/sample) was incubated with the primers (final concentration of 0.31 μM in RT reaction mixture) at 95°C for 5 min just before RT reaction to denature the viral dsRNA (69). RT was performed with PrimeScript RT enzyme at 50°C for 15 min (total, 10 μl/sample) followed by heat inactivation at 85°C for 5 s. The cDNA samples were treated with exonuclease I (2.5 U/sample) (TaKaRa; product number 2650A) at 37°C for 30 min followed by heat inactivation at 80°C for 15 min.

Real-time PCR was performed by using a TaKaRa TB green premix *Ex Taq* II (Tli RnaseH Plus). One microliter of exonuclease I-treated cDNA was added to a 19-μl reaction mixture containing primers +strandFluc_For and Tag primers (for detection of plus/positive strand viral RNA) or −strandFluc_Rev and Tag primers (for detection of minus/negative strand viral RNA) (final concentration of 0.8 μM each). The condition of real-time PCR consisted of a denaturation step at 95°C for 30 s and subsequent 40 cycles of thermal cycling of 95°C for 3 s and 60°C for 30 s. The fluorescence emission of the probe was monitored and analyzed using a 7500 fast real-time PCR system (Applied Biosystems).

### Time-lapse analysis of PV(EGFP)_pv_ infection.

RD(WT) cells or RD(Δ*PI4KB*) cells were seeded into a 35-mm dish (MatTek Corp.; part number P35GC-0-14-C) (8 × 10^3^ cells per dish in 1 ml of 10% FCS-DMEM) and then incubated at 37°C for 1 day. The dishes were set in a time-lapse fluorescence microscopy (Olympus, LCV110) and then infected with PV(EGFP)_pv_ at an MOI of 10. The cells were incubated at 37°C for 24 h. Phase-contrast and EGFP fluorescence images were collected every 15 min during the incubation and then analyzed with MetaMorph software (Molecular Devices, LLC.).

### Flow cytometry.

RD(WT) or RD(Δ*PI4KB*) cells (2.8 × 10^5^ cells) were infected with PV(EGFP)_pv_ at an MOI of 0.5 and then incubated at 37°C. Cells were collected at 8, 16, or 24 h p.i. and then stained with SYTOX red dead cell stain (1:1,000; Invitrogen; catalog number S34859) to detect membrane-compromised cells (dead cells) in 0.35 ml of 10% FCS-DMEM at 4°C for 20 min. About 5.0 × 10^4^ cells were measured per sample with a BD FACSCanto II flow cytometer (BD Biosciences). Data were analyzed using FlowJo software (FlowJo, LLC).

Cells were classified into 4 groups as follows:

Group 1 (infected cells with intact membrane): EGFP-positive and SYTOX red-negative cells

Group 2 (infected cells with compromised membrane [dead cells]): EGFP-positive and SYTOX red-positive cells

Group 3 (noninfected cells with intact membrane): EGFP-negative and SYTOX red-negative cells

Group 4 (noninfected cells with compromised membrane [dead cells]): EGFP-negative and SYTOX red-positive cells

Normalized EGFP signal per cell and population of infected cells with compromised membrane (%) were determined as below:

$$\text{Normalized EGFP signal per cell}=\left(\frac{\text{EGFP signals in Group}1\text{ cells}}{\text{EGFP signals in Group }3\text{ cells}}\right)$$

$$\text{Population of infected cells with compromised membrane }(\text{\%})=100\times \left(\frac{\text{Number of Group }2\text{ cells}}{\text{Number of Group }1\text{ cells}+\text{Number of Group }2\text{ cells}}\right)$$

### Statistical analysis.

Results of experiments are shown as means with standard deviations. Values of *P* < 0.05 by one-tailed *t* test were considered to indicate a significant difference and were indicated by asterisks (*, *P* < 0.05; **, *P *< 0.01; ***, *P *< 0.001). Multiple comparisons were conducted using the Tukey-Kramer method.

### Data availability.

Raw data sets not included in the manuscript or in the supplementary information are available from the corresponding author upon request.

## Supplementary Material

Reviewer comments
