# Supplementary material for: High-Order Epistasis and Functional Coupling of Infection Steps Drive Virus Evolution toward Independence from a Host Pathway
Source: Microbiol Spectr. 2021 Sep 1;9(2):e00800-21. doi: 10.1128/Spectrum.00800-21 (PMC8557862; doi:10.1128/Spectrum.00800-21)
Supplement: SUPPLEMENTAL FILE 5 — Supplemental material. Download SPECTRUM00800-21_Supp_5_seq3.docx, DOCX file, 2.3 MB. [file spectrum00800-21_supp_5_seq3.docx]

**Supporting information:**

Title: High-order に﷽﷽﷽﷽﷽﷽﷽﷽﷽﷽﷽﷽﷽﷽﷽法lowing primers。﷽﷽﷽﷽﷽﷽﷽﷽﷽﷽﷽﷽﷽﷽﷽﷽れをした）これは正確なタイタed sensitivity to PI4KB/OSBP inhibitors..eron response, sugge\\ons)onsnantcholesterol.tween Multi-tiereHighaepistasis and functional couplings of infection steps drive virus evolution toward independence from a host pathway

Author: Minetaro Arita

The number of videos: 4

The number of tables: 1

The number of figures: 4

**Video S1.** Time-lapse analysis of PV(EGFP)_pv_ infection in RD(WT) cells, set1.

**Video S2.** Time-lapse analysis of PV(EGFP)_pv_ infection in RD(WT) cells, set2.

**Video S3.** Time-lapse analysis of PV(EGFP)_pv_ infection in RD(Δ*PI4KB*) cells, set1.

**Video S4.** Time-lapse analysis of PV(EGFP)_pv_ infection in RD(Δ*PI4KB*) cells, set2.

|  | **Plus or minus-strand ssRNA of PV(Fluc) replicon**  (1.0×10^8^ copies/μL) | | | |
| --- | --- | --- | --- | --- |
| **Polarity of used RNA** | Plus | Plus | Minus | Minus |
| **Primers for RT and real-time PCR** | For plus strand detection | For minus strand detection | For plus strand detection | For minus strand detection |
| **Target of measurement** | Plus strand | Minus strand  (false-positive detection) | Plus strand  (false-positive detection) | Minus strand |
| **Detected number of copies of RNA per μL*** | 1.0×10^8^ | 7.5×10^4^  (4.8×10^3^) | 4.3×10^4^  (3.4×10^3^) | 1.0×10^7^  (1.6×10^5^) |
| **Relative specificity**** | 1.0 | 0.00075  (0.000048) | 0.0042  (0.00033) | 1.0 |

*Number of copies of plus-strand RNA with plus-strand detection in real-time PCR was taken as the standard.

** Number of copies of plus-strand RNA detected with primers for plus-strand detection or of minus-strand RNA detected with primers for minus-strand detection were taken as 1 for the relative specificity.

Standard deviation is shown in parentheses. *n* = 3.

**Table S1. Specificity of strand-specific real-time RT-PCR targeting firefly luciferase gene in PV(Fluc) replicon.**


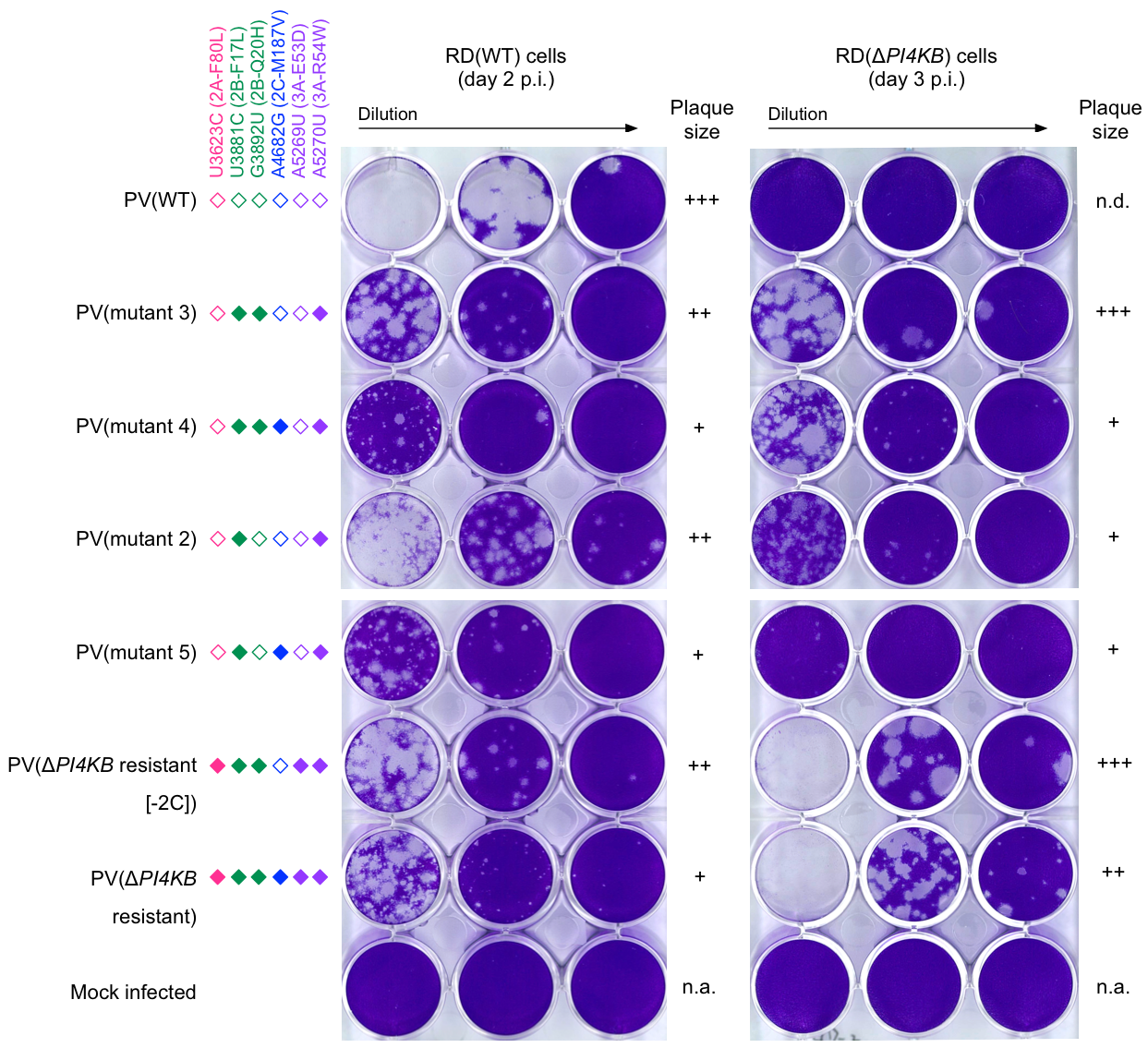


**Figure S1. Effect of the 2C-M187V mutation on viral spread in PI4KB/OSBP-independent infection.**

RD(WT) cells or RD(Δ*PI4KB*) cells were inoculated with diluted PV mutants, and then stained at 2 days p.i. or at 3 days p.i., respectively. Dilution of WT, mutant 3, and Δ*PI4KB* resistant [-2C] solutions were 1/10^5^, 1/10^6^, and 1/10^7^. Dilution of mutant 2, 4, 5, and Δ*PI4KB* resistant solutions were 1/10^4^, 1/10^5^, and 1/10^6^. Plaque phenotypes in the cells are shown. Filled diamonds represent nt mutations derived from PV1(Δ*PI4KB* resistant), and open diamonds represent nt of WT. The data are representative of two independent experiments.

**
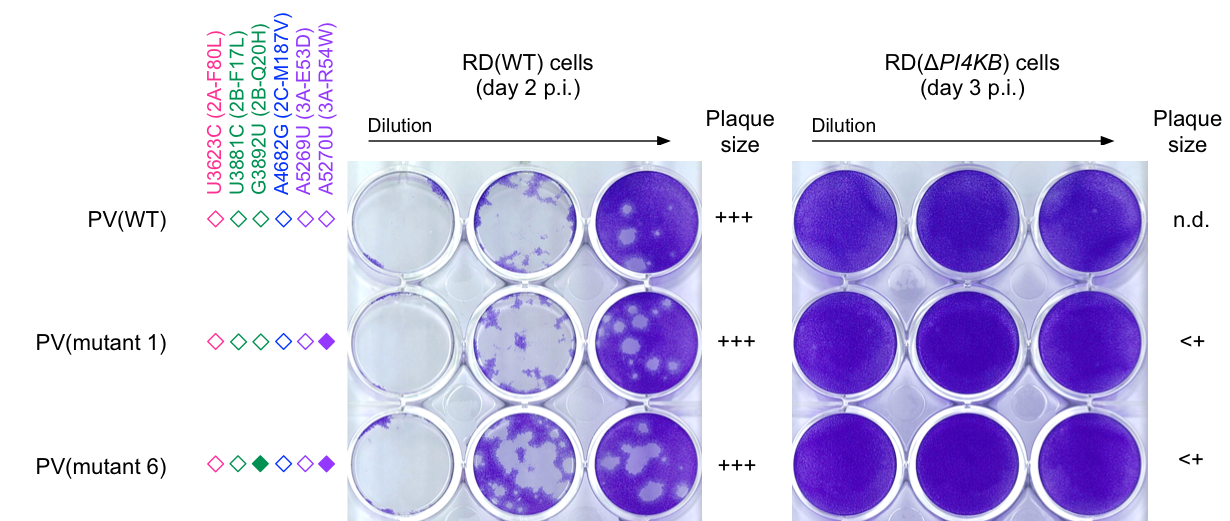
**

**Figure S2. Effect of the 2B-Q20H mutation on viral spread in PI4KB/OSBP-independent infection.**

RD(WT) cells or RD(Δ*PI4KB*) cells were inoculated with diluted PV mutants, and then stained at 2 days p.i. or at 3 days p.i., respectively. Dilution of WT, mutant 1, and 6 solutions were 1/10^4^, 1/10^5^, and 1/10^6^. Plaque phenotypes in the cells are shown. Filled diamonds represent nt mutations derived from PV1(Δ*PI4KB* resistant), and open diamonds represent nt of WT. The data are representative of two independent experiments.

**
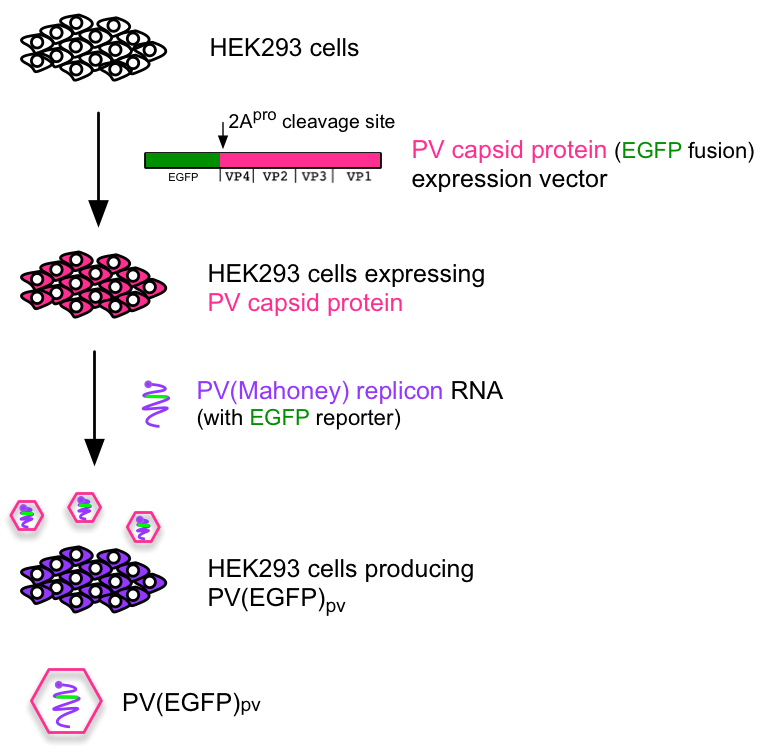
**

**Figure S3. Schematic view of PV(EGFP)_pv_ production.**

**
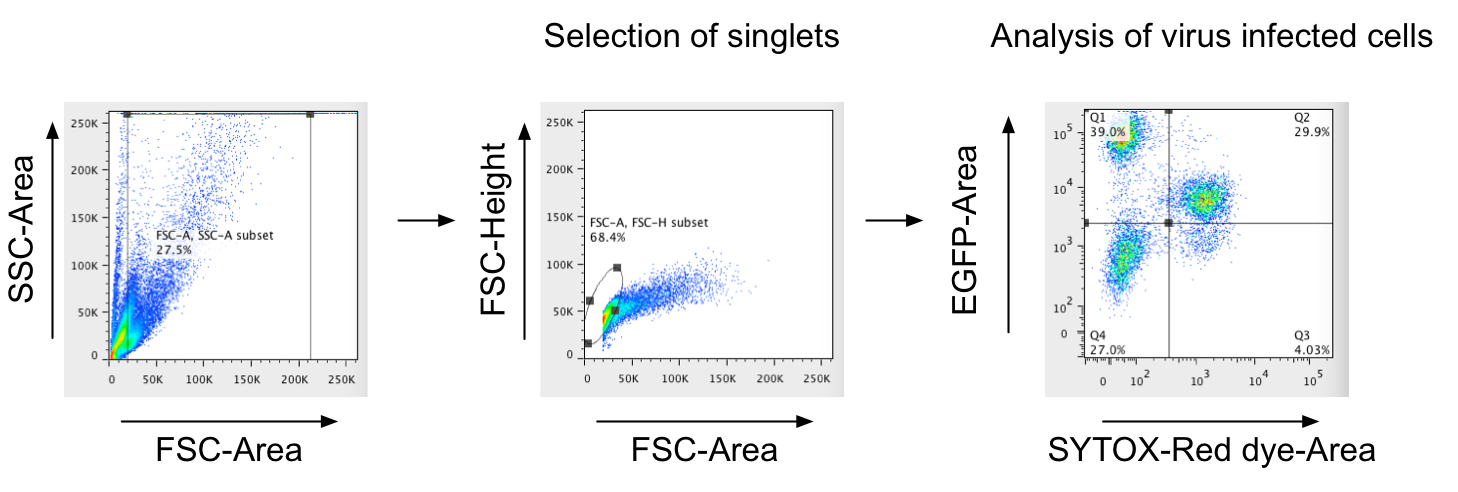
**

**Figure S4. Gating strategy of flow cytometry.**

Major population of the cells was selected by SSC-Area/FSC-Area window, and then singlets of the cells were selected in FSC-Height/FSC-Area window for quantification of virus replication level (EGFP reporter) and membrane-compromised cells (SYTOX-Red dye staining).
