# Supplementary material for: High-Order Epistasis and Functional Coupling of Infection Steps Drive Virus Evolution toward Independence from a Host Pathway
Source: Microbiol Spectr. 2021 Sep 1;9(2):e00800-21. doi: 10.1128/Spectrum.00800-21 (PMC8557862; doi:10.1128/Spectrum.00800-21)
Supplement: Reviewer comments [file reviewer-comments.pdf]

AMERICAN SOCIETY FOR MICROBIOLOGY | Microbiology Spectrum

High-order epistasis and functional couplings of infection steps drive virus evolution toward independence from a host pathway

Minetaro Arita

Corresponding Author(s): Minetaro Arita, National Institute of Infectious Diseases

**Review Timeline:**	Submission Date:	July 8, 2021
	Accepted:	August 4, 2021

Editor: Manjula Kalia

Reviewer(s): The reviewers have opted to remain anonymous.

Transaction Report:

(Note: With the exception of the correction of typographical or spelling errors that could be a source of ambiguity, letters and reports are not edited. The original formatting of letters and referee reports may not be reflected in this compilation.)

DOI: https://doi.org/10.1128/Spectrum.00800-21

August 4, 2021

Dr. Minetaro Arita
National Institute of Infectious Diseases
Department of Virology II
4-7-1 Gakuen, Musashimurayama-shi
Tokyo 208-0011
Japan

Re: Spectrum00800-21 (High-order epistasis and functional couplings of infection steps drive virus evolution toward independence from a host pathway)

Dear Dr. Minetaro Arita:

Your manuscript has been accepted, and I am forwarding it to the ASM Journals Department for publication. You will be notified when your proofs are ready to be viewed.

The ASM Journals program strives for constant improvement in our submission and publication process. Please tell us how we can improve your experience by taking this quick Author Survey.

As an open-access publication, Spectrum receives no financial support from paid subscriptions and depends on authors' prompt payment of publication fees as soon as their articles are accepted. You will be contacted separately about payment when the proofs are issued; please follow the instructions in that e-mail. Arrangements for payment must be made before your article is published. For a complete list of **Publication Fees**, including supplemental material costs, please visit our website.

Corresponding authors may join or renew ASM membership to obtain discounts on publication fees. Need to upgrade your membership level? Please contact Customer Service at Service@asmusa.org.

Thank you for submitting your paper to Spectrum.

Sincerely,

Manjula Kalia
Editor, Microbiology Spectrum

Journals Department
American Society for Microbiology
1752 N St., NW
Washington, DC 20036
E-mail: spectrum@asmusa.org

Video S4: Accept
Video S1: Accept

Video S3: Accept
Video S2: Accept
Table S1, Fig. S1,S2, S3, S4 : Accept
